# Whole genome sequencing of ceftolozane/tazobactam-resistant, XDR *Pseudomonas aeruginosa* ST773 in hospitalized critically ill infants and young children with ventilator-associated pneumonia

**DOI:** 10.3389/fcimb.2026.1870600

**Published:** 2026-07-10

**Authors:** Samira M. Hamed, Amira F.A. Hussein, Moshira H. Ezz El Arab, Mohamed H. Al-Agamy, Mohammed Aufy, Mohamed Abdelmoteleb, Mai M. Zafer

**Affiliations:** 1 Department of Microbiology and Immunology, Faculty of Pharmacy, October University for Modern Sciences and Arts (MSA), Giza, Egypt; 2 Clinical and Chemical Pathology Department, Faculty of Medicine, Cairo University, Cairo, Egypt; 3 Department of Pharmaceutics, College of Pharmacy, King Saud University, Riyadh, Saudi Arabia; 4 Department of Pharmaceutical Sciences, Division of Pharmacology and Toxicology, University of Vienna, Vienna, Austria; 5 Botany Department, Faculty of Science, Mansoura University, Mansoura, Egypt; 6 Department of Microbiology and Immunology, Faculty of Pharmacy, Ahram Canadian University, Cairo, Egypt

**Keywords:** Extensive drug resistance, NDM, *Pseudomonas aeruginosa*, resistance islands, ST773, ventilator-associated pneumonia, whole genome sequencing

## Abstract

**Introduction:**

Ventilator-associated pneumonia (VAP) caused by *Pseudomonas aeruginosa* poses a major therapeutic challenge for critically ill infants and young children.

**Methods:**

In this study, we assessed the antimicrobial susceptibility profiles of 42 *P. aeruginosa* isolates recovered from neonatal and paediatric intensive care unit (NICUs and PICU) patients with VAP between March and September 2021. Five isolates (11.9%) exhibited an extensively drug-resistant (XDR) phenotype and were resistant to both ceftazidime–avibactam and ceftolozane–tazobactam. These five isolates were subjected to whole-genome sequencing (WGS) and comparative genomic analyses.

**Results:**

All five isolates belonged to serogroup O11 and sequence type ST773. Nevertheless, WGS-based phylogenetic analyses, such as core genome MLST and SNP-based phylogeny, showed that the isolates were non-clonal and had a closer genetic relationship to previously identified ST773 strains from Egypt and Germany. Several multidrug efflux systems and a broad range of acquired antimicrobial resistance genes, such as *bla*NDM-1, *rmtB4, tet(G)*, and *flor2*, carried on a conserved integrative conjugative element previously reported in ST773, were found by genomic analysis. Furthermore, on a genomic island inserted downstream of the *glmS* gene, a class 1 integron containing *qnrVC1, aadA11, qacEΔ1*, and *sul1* was found. All strains possessed the same quinolone resistance-determining region alterations (*gyrA* T83I and *parC* S87L) and the same array of virulence-associated genes linked to motility, secretion systems, iron acquisition, quorum sensing, and toxin production.

**Discussion:**

This study reports the identification of non-clonal, XDR *P. aeruginosa* ST773 isolates associated with VAP in critically ill infants and young children. Despite their non-clonal nature, the isolates shared key features, including mobile genetic elements carrying important resistance genes and a consistent virulence gene profile. These findings highlight the clinical significance of this sequence type and raise concerns about its potential impact in the NICU and PICU settings. Continuous genomic surveillance, along with improved antimicrobial stewardship and stricter infection control practices, remains essential to limit its spread.

## 1 Introduction

Ventilator-associated pneumonia (VAP) is a serious healthcare-associated infection affecting high-risk patients receiving mechanical ventilation (Pu et al., 2024). In adult intensive care units (ICUs), up to 45% of patients being mechanically ventilated for more than five days may develop pneumonia, although the reported incidence varies depending on diagnostic criteria and patient characteristics (Papazian et al., 2020). VAP is associated with longer length of stay in the ICU, excessive use of broad-spectrum intravenous antibiotics, higher health care costs, and mortality rates ranging from 10% to 40% (Papazian et al., 2020). Similarly, infants and young children requiring intubation at the Neonatal or Paediatric Intensive Care Units (NICUs or PICUs) are also at risk of developing VAP. According to CDC criteria, the diagnosis of pneumonia in children under one year of age requires evidence of deteriorating gas exchange, radiographic abnormalities, and at least three clinical indicators (Bondarev et al., 2024) and the empirical therapy relies on local resistance patterns and patient-specific factors (Alriyami et al., 2022). Neonates, who are preterm or have low birth weight (<2500 g) or very low birth weight (<1500 g), are particularly vulnerable to VAP. Their underdeveloped immune systems, along with the need to use invasive devices like endotracheal tubes and central venous catheters, put them at high risk of developing this infection. In various locations, the incidence is reported to differ radically, with 1–63 episodes per 1000 ventilator days, which are indicative of both geographic disparities in infection control practices and differences in surveillance approaches (Rangelova et al., 2024).

*Pseudomonas aeruginosa* is a major opportunistic pathogen responsible for a wide range of healthcare-associated infections, particularly among critically ill and immunocompromised patients, including children. It is frequently encountered in ICUs and represents one of the leading causes of VAP, accounting for approximately 4% of reported cases (Kollef et al., 2014; Bhattacharya et al., 2023; Li et al., 2024a). Despite proper treatment, the mortality rate associated with VAP can be as high as 29.7%, specifically when caused by Gram-negative bacilli (Patil and Patil, 2017). Poor outcomes such as longer ventilation, more antibiotic exposure, and increased mortality compared with infection with susceptible strains are much more likely when infections are caused by multidrug-resistant (MDR) strains, which are more commonly reported in ICUs (Kula et al., 2020).

MDR *P. aeruginosa* is resistant to at least one agent from three or more classes that are generally effective against *P. aeruginosa.* Extensively drug-resistant (XDR) *P. aeruginosa* is defined as an isolate that remains susceptible to no more than two classes of antipseudomonal antimicrobial agents (Magiorakos et al., 2012). The concept of “difficult to treat resistance” (DTR) was introduced for isolates that are non-susceptible to important 1^st^ line anti-pseudomonal drugs (Kadri et al., 2018). DTR is considered more clinically relevant than other resistance definitions because it identifies isolates that are resistant to all first-line β-lactam agents and fluoroquinolones commonly used for empirical and targeted therapy. As a result, DTR more accurately reflects situations in which clinicians face severely limited treatment options and must rely on less effective, more toxic, or less well-validated alternative therapies.

The increase in MDR microorganisms triggering infections is growing worldwide and becoming more serious in developing countries, driven by inadequate antimicrobial stewardship programs, limited infection control resources, and insufficient surveillance systems (Hussein et al., 2024; Al-Ouqaili et al., 2025; Owaid and Al-Ouqaili, 2025; Amer et al., 2026). These trends are particularly concerning in infants and young children, for whom the range of safe and effective antimicrobial agents is more limited than in adults, thereby reducing treatment options and increasing the risk of adverse clinical outcomes.

*P. aeruginosa* can develop resistance in several ways, such as by decreasing the permeability of the outer membrane (e.g., loss of OprD), by producing and/or structurally modifying the chromosomal AmpC β-lactamase (PDC), upregulation of efflux pumps (e.g., MexAB-OprM), alteration of penicillin-binding proteins, and the acquisition of extended-spectrum β-lactamases (e.g., *bla_OXA_*_10_) (Lister et al., 2009; Wolter and Lister, 2013). Carbapenemase production, specifically *bla*_VIM_, plays a major role in carbapenem resistance globally, and is found in over 20% of carbapenem-resistant *P. aeruginosa* isolates (Zafer et al., 2015; Owaid and Al-Ouqaili, 2025).

The Infectious Diseases Society of America (IDSA) advises against using older β-lactam agents to treat isolates of *P. aeruginosa* with MDR and DTR phenotypes and instead recommends antimicrobial susceptibility testing (AST) of *P. aeruginosa* isolates against newer β-lactam agents, such as ceftolozane-tazobactam, ceftazidime-avibactam, imipenem-cilastatin-relebactam, and cefiderocol (Tamma et al., 2024). International surveillance studies indicate that ceftolozane-tazobactam and ceftazidime-avibactam have activity against ~76% and 74% of carbapenem-resistant *P. aeruginosa*, respectively, but with lower susceptibility rates in isolates from patients with cystic fibrosis (CF) (Sader et al., 2017; Castanheira et al., 2018; Sader et al., 2018).

Although there are numerous data on MDR, XDR, and DTR *P. aeruginosa* in adults in ICUs (Li et al., 2024a), there is a significant lack of information on resistance patterns, limitations in treatment, and clinical outcomes in paediatric VAP. Infections with ceftazidime-avibactam and ceftolozane-tazobactam-resistant isolates in infants and young children are extremely concerning due to their physiological vulnerabilities, limited antibiotic choices, and potential for drug toxicity.

Therefore, this study aimed to examine the antimicrobial resistance mechanisms and genomic characteristics of ceftazidime–avibactam and ceftolozane–tazobactam non-susceptible *P. aeruginosa* isolates recovered from critically ill infants and young children with VAP in the NICU and PICU. Employing whole-genome sequencing (WGS), we investigated the emergence of the high-risk clone ST773, its resistance determinants, mobile genetic elements, and virulence gene repertoire.

## 2 Materials and methods

### 2.1 Study design

This study focused on VAP in hospitalized critically ill infants and young children admitted to the NICU and PICU of the Faculty of Medicine, Cairo University, spanning the period from March 2021 to September 2021. The study population included patients aged 3 months to 5 years. The isolates were obtained from respiratory samples of 42 patients, with 13 of them experiencing infections caused by both *P. aeruginosa* and *Klebsiella pneumoniae*. The remaining 29 patients were identified to have infections solely attributed to *P. aeruginosa*. Only one *P. aeruginosa* isolate per patient was included in the study, the first clinically significant isolate meeting CDC VAP criteria.

The isolates were obtained from patients who were definitively diagnosed with VAP through the following criteria. VAP is characterized by the necessity of ventilation for more than 48 hours, along with either the initiation or modification of antibiotic therapy due to aggravated ventilation requirements (such as a rise in FiO_2_ by > 20%, increased pCO2, or heightened ventilation demand). Additionally, at least one of the following conditions must be met:

- Clinical deterioration, which includes temperature instability (axilla temperature > 37.5 °C or < 36.5 °C, tachycardia, hypotension, increased frequency of episodes with bradycardias and/or desaturations.
- New or worsening opacity, consolidation, or pleural effusion observed on chest X-ray.
- Changes in tracheal secretions.
- Abnormal laboratory parameters, such as C-reactive protein (CRP) > 10 mg/L, leucocytosis (white cell count > 20 × 10^9^/L), or leucopenia (white cell count < 5 × 10^9^/L).

A VAP case is confirmed when the above criteria are fulfilled, and the airway aspirate reveals the presence of a pathogenic microorganism. Samples recovered from critically ill infants and young children who met the complete CDC diagnostic criteria for VAP, including clinical deterioration, radiographic abnormalities, and abnormal inflammatory markers were included in the study, whereas isolates obtained in the absence of these criteria were considered colonizers and excluded from the study. Although both BAL and ETA samples were included, all isolates were interpreted within the clinical context of confirmed VAP, and only samples obtained from critically ill infants and young children fulfilling the complete CDC diagnostic criteria were considered. In our study, only the *P. aeruginosa* isolates were included.

Antimicrobial resistance phenotypes were classified following the international criteria recommended by Magiorakos et al. (2012). MDR *P. aeruginosa* was defined as non-susceptibility to at least one agent in ≥3 antimicrobial categories. XDR isolates were defined as non-susceptible to all but ≤2 antimicrobial categories. Besides, the DTR phenotype was defined according to Kadri et al. (2018) as non-susceptibility to all first-line, high-efficacy antipseudomonal agents, comprising β-lactams (piperacillin–tazobactam, ceftazidime, cefepime, aztreonam, meropenem, imipenem) and fluoroquinolones. These definitions were employed to all obtained isolates, and the five isolates selected for WGS were those demonstrating XDR phenotypes and reduced susceptibility to both ceftolozane–tazobactam and ceftazidime–avibactam, and additionally met the DTR criteria due to resistance to all standard first-line antipseudomonal agents.

### 2.2 Bacterial identification

*P. aeruginosa* isolates recovered from bronchoalveolar lavage (BAL) and endotracheal aspirate (ETA) samples were identified using a combination of conventional microbiological methods. The preliminary identification was based on Gram staining, colony morphology, and oxidase testing, followed by growth on cetrimide agar. Final confirmation was performed using VITEK^®^ 2 Compact system (bioMérieux).

### 2.3 Antimicrobial susceptibility testing

Antimicrobial susceptibility testing of the isolates was done using the disk diffusion method according to standard laboratory procedures. The susceptibility profiles against a panel of antimicrobial agents, including amoxicillin–clavulanic acid, cefoxitin, trimethoprim–sulfamethoxazole, ceftazidime, cefotaxime, imipenem, meropenem, ciprofloxacin, levofloxacin, amikacin, gentamicin, and piperacillin–tazobactam, were determined. Interpretation of inhibition zone diameters was carried out according to the Clinical and Laboratory Standards Institute (CLSI) guidelines (CLSI, 2024).

In addition, ceftazidime-avibactam and ceftolozane-tazobactam E-test strips (0.016-256 µg/ml) were obtained from bioMérieux (Marcy-I ‘Etoile, France) to assess the minimum inhibitory concentration (MIC) in accordance with the manufacturer’s instructions. The MIC results were interpreted using the breakpoints recommended by the CLSI for all the studied antimicrobials (CLSI, 2024).

### 2.4 WGS and bioinformatic analysis

Of the recovered *P. aeruginosa* isolates, five isolates were selected for whole-genome sequencing based on their antimicrobial susceptibility profiles, as they were the only isolates demonstrating reduced susceptibility to both ceftolozane–tazobactam and ceftazidime–avibactam.

Genomic DNA was extracted from all isolates using the QIAGEN DNA Purification Kit (Qiagen, Valencia, CA, USA). Libraries were prepared with the Nextera DNA Sample Preparation Kit (Illumina, USA), followed by WGS on the Illumina MiSeq platform generating paired-ends reads of 150 bp length. Sequencing depth of different samples ranged between 7.19x to 15.7x Quality of sequencing reads was evaluated using FastQC (Brown et al., 2017), followed by cleaning of low-quality bases using Trimmomatic (Bolger et al., 2014). SPAdes assembler was integrated for *de novo* genome assembly (Bankevich et al., 2012) using multiple K-mers (33, 55, 77, and 99). Quality of genome assembly was checked using QUAST (Gurevich et al., 2013). In addition, CheckM v1.0.18 was used to assess genome completeness and contamination (Parks et al., 2015) based on the presence and absence of lineage-specific marker genes following the default lineage_wf workflow. FastANI v1.34 was implemented to confirm the identity of our isolates through computing average nucleotide identity analysis (ANI) of the five genomes against the reference *P. aeruginosa* strain HPA0124 (GenBank accession: CP137504.1) (Jain et al., 2018). The draft genomes underwent annotation via the NCBI Prokaryotic Genome Annotation Pipeline (PGAP) (Tatusova et al., 2016).

### 2.5 Strain typing

Strain typing and epidemiological analysis were performed on our five *P. aeruginosa* isolates alongside similar genomes retrieved from the Bacterial and Viral Bioinformatics Resource Center (BV-BRC) database (accessed 6 January 2026; https://www.bv-brc.org/) (**Supplementary Table 1**). Similar genomes were identified using the Similar Genome Finder service (Shukla et al., 2025). Four in silico typing approaches were employed, including: serotyping with PAst, multilocus sequence typing (MLST), core-genome MLST (cgMLST), and SNP-based phylogenetic analysis.

#### 2.5.1 In silico serotyping

In silico serotyping was performed on the draft genomes using the *Pseudomonas aeruginosa* serotyper (PAst) hosted by the Center for Genomic Epidemiology available at: https://www.genomicepidemiology.org/services/. The serotyper assigns *P. aeruginosa* isolates to one of 11 serogroups through BLAST-based analysis of the O-antigen synthesis gene (OSA) cluster extracted from WGS data.

#### 2.5.2 Multilocus sequence typing

The sequence types (STs) of all strains were determined using fastMLST v0.0.16 (Guerrero-Araya et al., 2021). Assembled genomes were queried against the *P. aeruginosa* MLST typing database (accessed on 7 January 2026), and exact matches across all loci were used to assign the corresponding ST to each isolate.

#### 2.5.3 Core genome multilocus sequence typing

cgMLST analysis was performed using ChewBBACA, with adaptation of the *P. aeruginosa* Ridom cgMLST scheme (accessed 7 January 2026; https://www.cgmlst.org/ncs), which comprises 3,867 genes. Of these, 3,796 genes were present in ≥95% of our collection (including study isolates and BV-BRC similar genomes) and thus included in the final cgMLST analysis. A minimum spanning tree (MST) was generated from the cgMLST results using PHYLOViZ 2.0 (Francisco et al., 2012), to visualize the genetic relatedness and allelic differences among the studied isolates and the closest global strains.

#### 2.5.4 SNP-based phylogeny

SNP-based phylogenetic analysis was conducted using CSI Phylogeny 1.4 (Kaas et al., 2014) on our five *P. aeruginosa* study isolates alongside 36 similar genomes retrieved from BV-BRC. *P. aeruginosa* NCTC13715 (Accession: LR134330) served as the reference genome. The resulting tree was visualized in the Interactive tree of life (iTOL) tool v6 (Tatusova et al., 2016).

### 2.6 Detection of antimicrobial resistance genes, virulence genes, and associated mobile genetic elements

AMR genes across all genomes (five study isolates and 36 BV-BRC references) were identified using NCBI AMRFinder v4.2.5 (Feldgarden et al., 2019) with default parameters. The Virulence Factor Database (VFDB) was used to predict virulence genes carried by the test strains (Chen et al., 2005). MGEs associated with AMR and virulence genes were identified using MobileElementFinder (v1.0.3) (Johansson et al., 2021).

To investigate the genomic context of AMR genes, reference-based mapping was performed against the complete genome of *P. aeruginosa* ST773 (GenBank accession: CP041945). This reference was chosen because of its high-quality and its close phylogenetic relatedness to the study isolates. Raw sequencing reads were aligned using BWA-MEM (v0.7.17) (Li and Durbin, 2010), and the resulting alignments were sorted and processed with SAMtools (v1.17) (Li et al., 2009). Variant calling was executed through the BCFtools (v1.17) suite, utilizing the mpileup and call functions to identify high-confidence genomic variations. To ensure analytical rigor, variant sites were filtered for quality (QUAL > 30) before generating final consensus FASTA sequences using bcftools consensus (Li, 2011).

## 3 Results

### 3.1 Bacterial isolates

A total of 42 P*. aeruginosa* isolates were recovered from BAL fluid and ETA specimens collected from 42 infants and young children with confirmed VAP, diagnosed according to the criteria outlined in the Materials and Methods. Thirteen patients (31%) had mixed infections involving both *P. aeruginosa* and *K. pneumoniae*, while 29 (69%) had *P. aeruginosa* mono-infections. Overall, 17 patients (40%) died, and 25 (60%) survived. It is noteworthy to mention that mortality rates in this group of patients could not be attributed exclusively to infection with MDR/XDR *P. aeruginosa* as many patients had significant underlying conditions that likely contributed to clinical outcomes. Therefore, the reported mortality reflects the overall clinical complexity of critically ill NICU and PICU patients.

### 3.2 Antimicrobial susceptibility profiles

**Figure 1:**
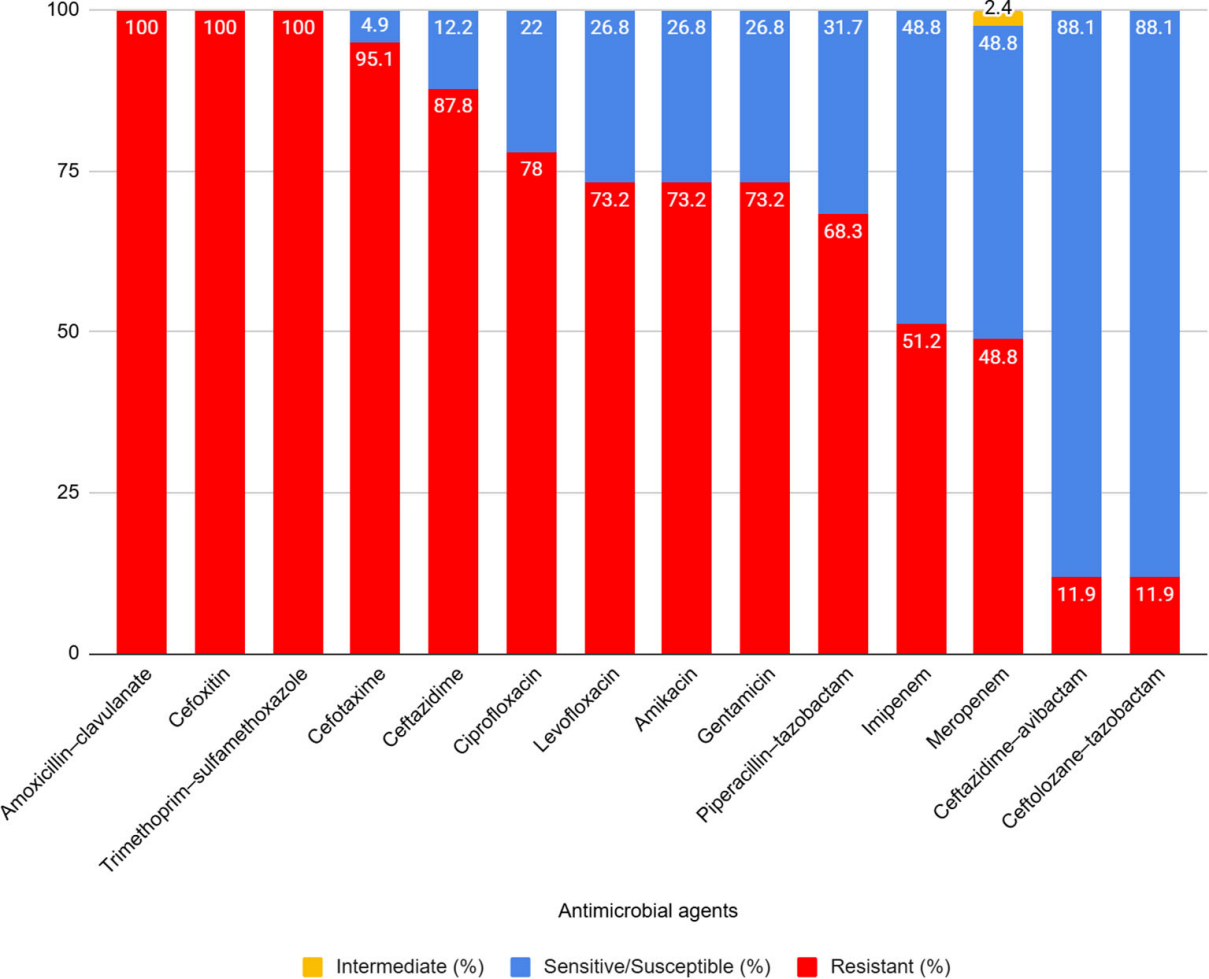
Antimicrobial susceptibility profiles of the *P. aeruginosa* isolates included in this study. Stacked bars show the percentage of isolates classified as resistant, susceptible, or intermediate to each antimicrobial agent according to susceptibility testing results. Antibiotics are arranged in descending order based on the proportion of resistant isolates.

The antimicrobial susceptibility profile of the *P. aeruginosa* isolates included in the current study demonstrated high levels of resistance to most of the antimicrobial agents tested. Complete resistance was observed to amoxicillin–clavulanate, cefoxitin, and trimethoprim–sulfamethoxazole. High resistance rates were also detected against third-generation cephalosporins, fluoroquinolones, and aminoglycosides, while nearly half of the isolates were resistant to carbapenems. Piperacillin–tazobactam retained moderate activity against the isolates. The MICs of ceftazidime–avibactam and ceftolozane–tazobactam were determined exclusively using E-test strips. Five of the 42 P*. aeruginosa* isolates (11.9%) were resistant to both ceftazidime–avibactam and ceftolozane–tazobactam. These isolates exhibited XDR phenotypes according to internationally accepted criteria. The percentages of resistance to all tested antimicrobial agents are presented in **Figure 1**.

### 3.3 Draft genome features

Genome assemblies of the five isolates yielded between 134 and 316 contigs, with total genome sizes ranging from 6,808,630 bp to 6,878,919 bp and a mean G+C content of 66 %. All genomes were of high quality with approximately >98% completeness and <1% contamination). Post-assembly and annotation metrics of the isolates are shown in **Supplementary Table 2**. Four or five rRNA genes, one transfer-messenger RNA (tmRNA), and about 61 to 62 tRNA copies were predicted to be encoded within the genomes. All isolates were confirmed to be *P. aeruginosa* by ANI analysis against the genome of the reference type strain with >99% ANI.

### 3.4 Comparative strain typing and evolutionary relationship of the isolates

**Figure 2:**
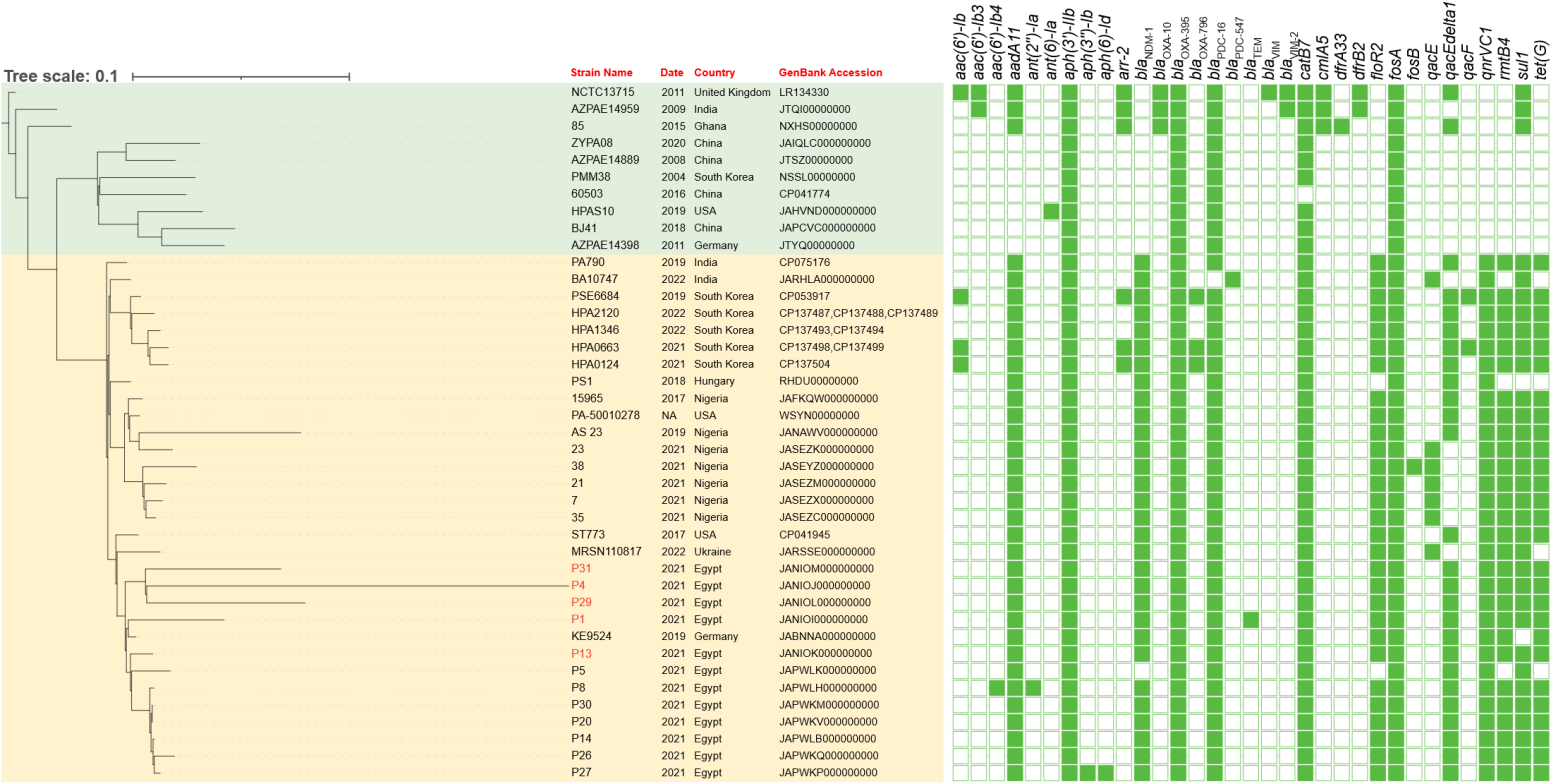
SNP-based phylogenetic tree illustrating the relationships among the five isolates from this study (names in red) and global *P. aeruginosa* ST773 strains. The *bla*_NDM_-positive ST773 cluster is highlighted in yellow, and the *bla*_NDM_-negative cluster is highlighted in green. Resistance gene profiles are indicated by solid green squares (positive) and hollow squares (negative). All strains were isolated from human sources, except AS_23, which originated from soil.

All five isolates collected in this study belonged to serogroup O11. MLST analysis predicted the following alleles: *acsA* (5), *aroE* (4), *guaA* (5), *mutL* (5), *nuoD* (5), *ppsA* (7), and *trpE* (8), corresponding to sequence type ST773. SNP-based phylogenetic analysis revealed that our isolates clustered closely, with pairwise SNP differences ranging from 64 to 251, as shown in **Figure 2**. These isolates also clustered with a strain collected in Germany in 2019 (KE9524) and with strains from Egypt collected in 2021. Notably, pairwise SNP differences between our isolates and KE9524 ranged from 18 to 197, while differences with the Egyptian strains ranged from 20 to 214.

**Figure 3:**
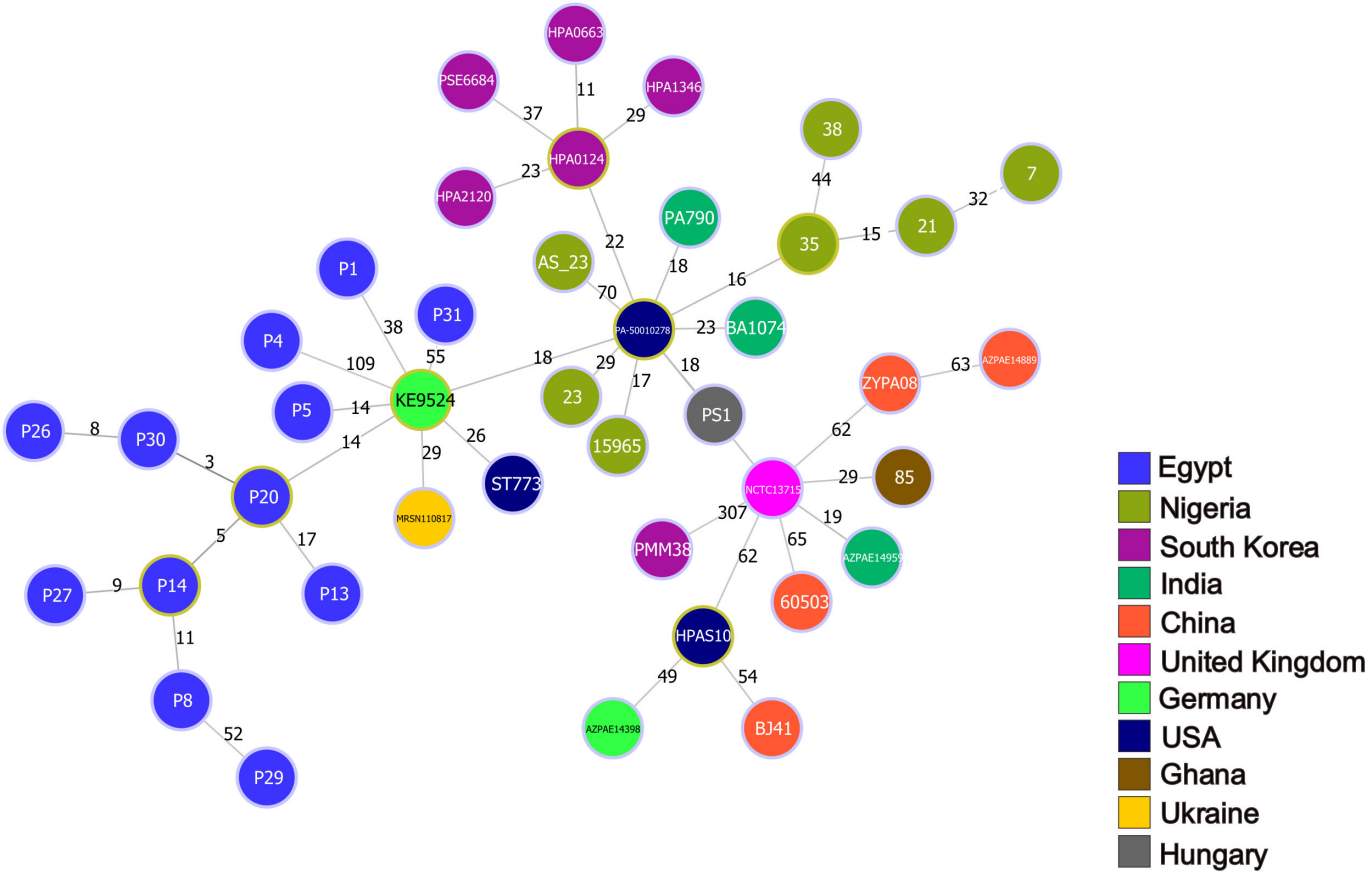
MST based on cgMLST profiles of the study isolates and the closest global strains. Each node represents a single strain, and branch labels indicate the number of allelic differences between directly connected strains. The tree highlights the genetic relationships among the isolates, showing that the study strains are more closely related to Egyptian isolates and the German strain KE9524 than to each other, in agreement with the SNP-based phylogenetic analysis.

This observation was further supported by cgMLST analysis (**Figure 3**), which showed that our isolates shared fewer allelic differences with previously reported Egyptian strains and the German strain KE9524 than with each other.

### 3.5 Antimicrobial resistance genes

Genome analysis of the five *P. aeruginosa* strains revealed the presence of multiple multidrug (MD) efflux pumps, including MexAB-OprM, MexCD-OprJ, MexEF-OprN, MexGHI-OpmD, MexJK-OprM, MexPQ-OpmE, MexXY-OprM, MexVW-OprM, and MuxAB-OpmB/MuxC. In addition, the strains harbored acquired resistance genes conferring resistance to a broad range of antibiotic classes, including carbapenems, aminoglycosides, fluoroquinolones, fosfomycin, monobactams, cephalosporins, phenicols, sulfonamides, and tetracyclines, as summarized in **Table 1**. The genomes also carried genes that encode disinfectant-exporting efflux pumps, specifically the triclosan efflux pump TriABC-OpmH and the quaternary ammonium compound efflux pump QacE delta 1.

**Table 1:** Antimicrobial resistance determinants carried by the five isolates.

Drug class	Resistance mechanism	Resistance determinant
Carbapenems	antibiotic inactivation	*bla*_NDM-1_/*bla*_OXA-395_
Aminoglycosides	antibiotic inactivation	*aadA11/aph(3’)-IIb*
	antibiotic efflux	*emrE*
	antibiotic target alteration	*rmtB4*
Fluoroquinolones	antibiotic target alteration	*gyrA* T83I*/parC* S87L
	antibiotic target protection	*qnrVC1*
Fosfomycin	antibiotic inactivation	*fosA*
Monobactam/carbapenem/cephalosporin	antibiotic inactivation	*bla*_PDC-16_
Phenicols	antibiotic inactivation	*catB7*
	antibiotic efflux	*cmlA9 (flor2)*
Sulfonamides	antibiotic target replacement	*sul1*
Tetracyclines	antibiotic efflux	*tet(G)*

In addition to intrinsic chromosomal resistance genes, including *bla*_OXA-395_, *bla*_PDC-16_, *aph(3′)-IIb*, *catB7*, and *fosA*, several acquired AMR genes were identified in association with MGEs.

Mapping the sequencing reads from all clinical isolates against the *bla*_NDM_-harboring ICE of *P. aeruginosa* ST773 reference (GenBank accession: CP041945), previously reported by Pitart et al. (2025), revealed sequence conservation. Read alignments demonstrated that the sequence identity across all studied isolates is at least 99.93%. Other resistance genes carried by the ICE included: *rmtB4*, *tet(G)*, and *flor2*.

**Figure 4:**
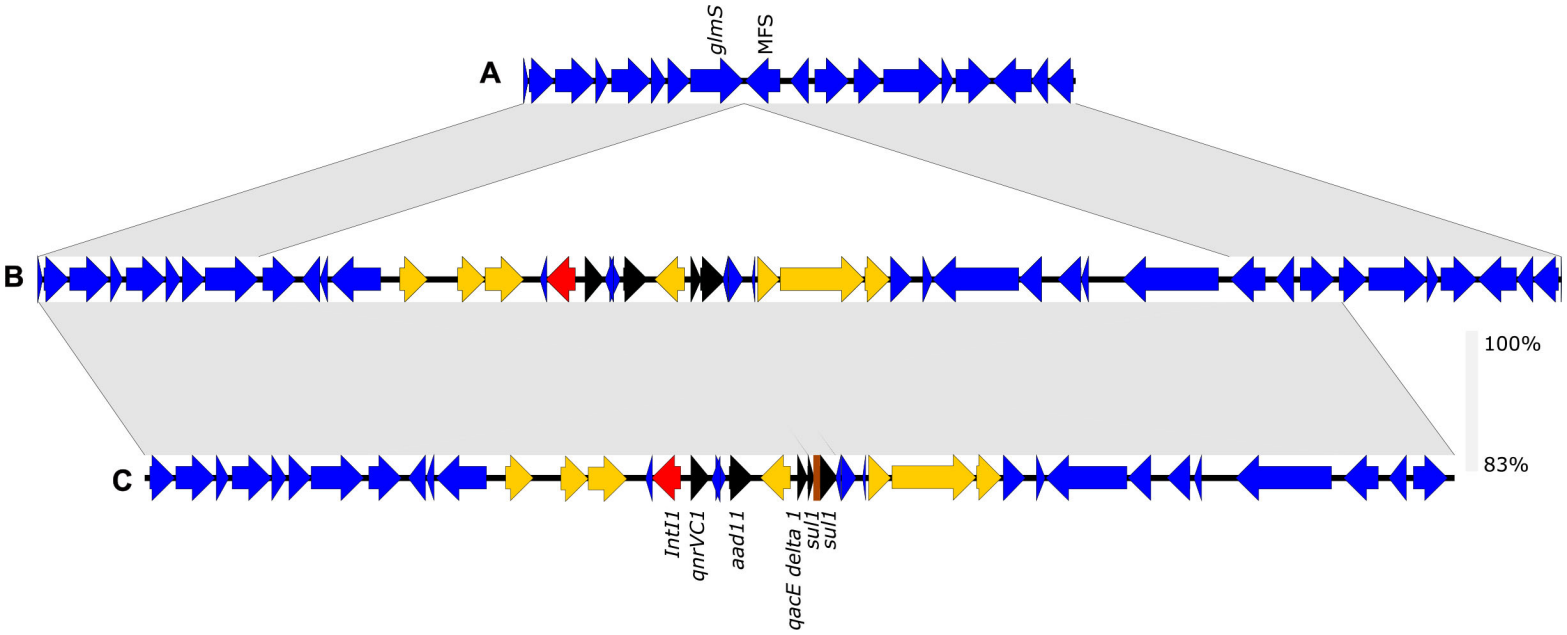
Gene maps showing comparative analysis of the genomic island carrying the class 1 integron and associated AMR genes. Panel A shows *P. aeruginosa* strain 60503 (GenBank accession: CP041774; region 6,775,900 – 6,795,088), Panel B shows *P. aeruginosa* ST773 (GenBank accession: CP041945; region 6,768,340 – 6,821,303), and Panel C shows *P. aeruginosa* isolate P4 sequenced in the current study (nodes 4 and 16). The grey blocks indicate regions of pairwise sequence similarity. Arrows represent open reading frames, with yellow arrows indicating transposase-coding genes, the red arrow indicating the integrase-coding gene, and black arrows representing AMR genes. The brown rectangle indicates an assembly gap. The gene *glmS* is a glutamine-fructose-6-phosphate transaminase-coding gene. MFS is a major facilitator superfamily efflux pump-coding gene.

All four resistance genes (*qnrVC1*, *aadA11*, *qacE delta1*, and *sul1*) were co-localized within a class 1 integron on a single contig. Pairwise comparative genomic analysis was conducted to characterize the complete genetic element carrying these genes and to determine its genomic insertion site. The integron-containing region was compared between two *P. aeruginosa* strains belonging to ST773 with complete genome sequences available in the NCBI database. One strain, *P. aeruginosa* strain ST773 (GenBank accession: CP041945), harbored the resistance genes, whereas the second strain, *P. aeruginosa* strain 60503 (GenBank accession: CP041774), lacked these resistance determinants. This comparison demonstrated that the resistance genes and their associated class 1 integron were located on a genomic island inserted downstream of the *glmS* gene, which encodes glutamine–fructose-6-phosphate transaminase. **Figure 4** shows gene maps demonstrating the pairwise comparison between *P. aeruginosa* strain 60503, *P. aeruginosa* strain ST773, and isolate P4.

### 3.6 Virulence genes

To further characterize the isolates’ virulence potential, virulence gene analysis was performed using VFDB. All five isolates exhibited an identical virulence gene profile. The detected virulence genes were classified into functional categories, including motility, adherence, immune evasion, antimicrobial activity, iron acquisition, secretion systems, quorum sensing, and toxin production. As shown in **Table 2**.

**Table 2:** Virulence gene profile of the five *P. aeruginosa* isolates.

VF Class	VF	Genes
Motility	Flagella	*fleNQRS*
		*flgABCDEFGHIJKLMN*
		*flhABF*
		*fliACDEFGHIJKLMNOPQRST*
		*motABCDY*
	LPS O-antigen	*--*
	Type IV pili biosynthesis	*fimUV*
		*pilBCDEFMNOPQRSTUVXYZ*
	Type IV pili twitching motility related proteins	*chpABCDE*
		*pilGHIJK*
Antimicrobial activity	Phenazines biosynthesis	*phzABCEDEFGHMS*
Antiphagocytosis	Alginate biosynthesis	*alg44*
		*alg8*
		*algACDEFGIJKLX*
	Alginate regulation	*algP/algR3-algQRUWZ*
		*mucABCDEP*
Biosurfactant	Rhamnolipid biosynthesis	*rhlABC*
Enzyme	Hemolytic phospholipase C	*plcH*
	Non-hemolytic phospholipase C	*plcN*
	Phospholipase C	*plcB*
	Phospholipase D	*pldA*
Iron uptake	Pyochelin receptor	*fptA*
	Pyochelin	*pchABCDEFGHIR*
	Pyoverdine receptors	*fpvA*
	Pyoverdine	*pvdAEFGHIJLMNOPQRSTY*
Protease	Alkaline protease	*aprA*
	Elastase	*lasAB*
	Protease IV	*prpL*
Quorum sensing	Acylhomoserine lactone synthase	*hdtS*
	N-(3-oxo-dodecanoyl)-L-homoserine lactone QS system	*lasIR*
	N-(butanoyl)-L-homoserine lactone QS system	*rhlIR*
Regulation	GacS/GacA two-component system	*gacSA*
Secretion system	Hcp secretion island-1 encoded type VI secretion system (H-T6SS)	*clpV1*
		*fha1*
		*hcp1*
		*icmF1*
		*ppkA*
		*pppA*
		*vgrG1*
	*P. aeruginosa* TTSS translocated effectors	*exoTUY*
	*P. aeruginosa* TTSS	*exsABCDE*
		*pcr1234DGHRV*
		*popBDN*
		*pscBCDEFGHIJKLNOPQRSTUXY*
Toxin	Exotoxin A (ETA)	*toxA*
	Hydrogen cyanide production	*hcnABC*

## 4 Discussion

In this study, we characterized the emergence of XDR *P. aeruginosa* ST773 in neonatal VAP in a tertiary hospital in Egypt. These isolates were not only resistant to older antipseudomonal agents but also to the newer β-lactam/β-lactamase inhibitor combinations, namely ceftolozane–tazobactam and ceftazidime–avibactam. Additionally, they harbored multiple antibiotic resistance determinants and a broad virulence gene repertoire, revealing the high-risk nature of this lineage.

The ICU environment is one of the most dynamic environments where the development of antimicrobial resistance can be observed as infections with resistant pathogens can have significant clinical and economic consequences (Alnimr, 2023; Abbas et al., 2026). The antimicrobial susceptibility findings of the current study revealed alarming resistance rates to most antipseudomonal agents used in NICUs and PICUs, in line with national trends in Egypt (Edward et al., 2023; Abdulhak et al., 2025b). These results reveal a worrying resistance landscape in NICUs and PICUs, where therapeutic options are already restricted for critically ill infants and young children. This resistance profile displays the combined association of intrinsic barriers, efflux activity, and acquired resistance determinants that characterize *P. aeruginosa* (Pang et al., 2019). The universal resistance to amoxicillin–clavulanate, cefoxitin, and trimethoprim–sulfamethoxazole detected in our isolates is in line with the organism’s intrinsic resistance characteristics. Furthermore, the high resistance rates to third-generation cephalosporins, fluoroquinolones, and aminoglycosides highlight the cumulative effects of long-term antibiotic exposure in critically ill infants and young children. These findings are consistent with studies reporting MDR *P. aeruginosa* in ICUs (Hafiz et al., 2023). The most worrying observation was the significant decrease in carbapenem susceptibility, with approximately half of the isolates resistant to imipenem and meropenem. This shows valuable clinical implications, given that current VAP treatment guidelines focus on carbapenems, piperacillin–tazobactam, cefepime, or ceftazidime as first-line antipseudomonal agents (Eid et al., 2025).

More recently, with the advent of new β-lactam/β-lactamase inhibitor combinations as ceftolozane–tazobactam and ceftazidime–avibactam, the IDSA proposed the term DTR to describe *P. aeruginosa* isolates that are resistant to all preferred first-line antipseudomonal agents (Tamma et al., 2024). In the present study, resistance to both ceftolozane–tazobactam and ceftazidime–avibactam was detected in 11.9% of *P. aeruginosa* isolates. These isolates demonstrated an XDR phenotype. This complex resistance profile closely aligns with the IDSA definition of DTR *P. aeruginosa*.

Interestingly, the five XDR isolates were serogroup O11 and sequence type ST773- a lineage that has gained increasing recognition as a high-risk clone with widespread antimicrobial resistance and global dissemination. This finding is consistent with global and Egyptian surveillance data showing that ST773 frequently carries *bla_NDM-1_* and demonstrates reduced susceptibility to newer β-lactam/β-lactamase inhibitor combinations (Jung et al., 2024; Pappa et al., 2024; Salem et al., 2024; Abdulhak et al., 2025a).

Although all isolates belonged to the same sequence type (ST773) and serogroup, SNP-based phylogenetic and cgMLST analyses indicated that they were likely non-clonal. Previous studies have suggested that *P. aeruginosa* isolates are generally considered part of the same outbreak when they differ by approximately 15–25 SNPs, with a mean genetic distance of 12.7 SNPs among outbreak-related strains (Romano-Bertrand et al., 2025). Similarly, Graña-Miraglia et al. (2026) proposed a pairwise genetic distance threshold of 24 SNPs as the maximum expected divergence between clonal strains. The pairwise SNP distances observed among our isolates exceeded these commonly accepted thresholds, supporting the conclusion that they were likely independently acquired or introduced rather than representing the dissemination of a single outbreak strain. This pattern suggests the circulation and repeated introduction of ST773 strains into the hospital setting and highlights the multifaceted epidemiology of *P. aeruginosa* in NICUs and PICUs.

WGS showed a dramatic overlap of resistance mechanisms in the ST773 isolates. Besides a complete set of intrinsic resistance determinants and multidrug efflux systems, all isolates contained the carbapenemase gene *bla*_NDM-1_, along with *rmtB4*, *tet(G)*, and *flor2*, located in a highly conserved ICE. This genomic configuration mirrors previous reports describing an NDM-positive ST773 lineage with broad resistance phenotypes and epidemic potential (Hernández-García et al., 2024; Jung et al., 2024). Such studies have suggested the presence of two large ST773 lineages, NDM-positive and NDM-negative, the former having a wider range of resistance phenotypes and epidemic potential. Our isolates are obviously located in the NDM-positive lineage, which supports the idea that this ICE is an important contributor to multidrug resistance and clonal success (Jung et al., 2024).

A class 1 integron harboring *qnrVC1*, *aadA11*, *qacEΔ1*, and *sul1* has been detected within a genomic island inserted downstream of the *glmS* gene. Class 1 integrons are well-recognized platforms for the capture, accumulation, and co-expression of gene cassettes encoding resistance to multiple antimicrobial classes, particularly aminoglycosides, sulfonamides, and quinolones. Their association with transposons and genomic islands further facilitates horizontal transfer and stable chromosomal integration, enabling long-term persistence of resistance determinants within successful clonal lineages (Shahcheraghi et al., 2010). The expression of a plasmid-mediated quinolone resistance gene, *qnrVC1*, initially identified in *Vibrio cholerae*, underscores the importance of horizontal gene transfer in shaping fluoroquinolone resistance in ST773, while the co-localization of *qacEΔ1* raises concerns about disinfectant-driven selective pressure in NICU environments (Kadry et al., 2017). Similar integron structures ST773 have been reported in other epidemic ST773 isolates, highlighting their role in fluoroquinolone resistance and decreased sensitivity to various antibiotic classes (Shahcheraghi et al., 2010; Belotti et al., 2015; Amer et al., 2026).

Although all ST773 isolates in this study showed high antimicrobial resistance, a complete and broad virulence gene repertoire was conserved. This preservation of virulence despite extensive resistance mirrors observations in other high-risk *P. aeruginosa* clones, suggesting that of comprehensive resistance determinants alongside intact virulence systems, such as type III and type VI secretion systems, quorum sensing and iron acquisition pathways, increases the potential of ST773 to survive in the hospital setting and induce serious infections especially in immunologically immature infants and young children. Numerous of these genes have direct implications for persistence and VAP pathogenesis in critically ill paediatric patients. The T3SS plays a critical role in immune evasion and tissue damage, delivering key exotoxins—*ExoA*, *ExoS*, *ExoU*, *ExoT*, and *ExoY*—that disrupt host cell signaling, inhibit protein synthesis, and mediate epithelial cell injury—mechanisms that are particularly damaging in infants with immature innate immunity (Derakhshan et al., 2021). Similarly, the Type VI secretion system (T6SS) is a well-known bacterial protein secretion machines that inject toxic effector proteins into nearby cells, thus facilitating both bacterial competition and virulence. It contributes to interbacterial competition and niche dominance within the endotracheal tube and lower airway, enabling persistent colonization (Colautti et al., 2025). The widespread repertoire of motility and adherence genes (flagellar operons, type IV pili, and twitching motility systems) allowing *P. aeruginosa* to attach to and colonize surfaces, augments biofilm formation on endotracheal tubes. Furthermore, the presence of quorum-sensing systems (*las*, *rhl*, *pqs*); a cell density-based intercellular communication system, which plays a vital role in regulation of the bacterial virulence and biofilm formation and iron acquisition pathways (pyoverdine and pyochelin biosynthesis) supports metabolic adaptability and coordinates the expression of virulence factors and biofilm formation under nutrient-limited conditions typical of the airway environment (Lee and Zhang, 2015). These findings are consistent with previous reports (Aroca Molina et al., 2024).

In conclusion, our findings reveal the emergence of non-clonal XDR/DTR *P. aeruginosa* ST773 in NICU and PICU patients. The success of this high-risk clone appears to be driven by the combination of extensive antimicrobial resistance, the acquisition of conserved mobile genetic elements, and the retention of key virulence determinants. The resistance to last-line β-lactam/β-lactamase inhibitor combinations highlights the urgent demand for ongoing genomic monitoring, enhanced infection control, and routine susceptibility testing to novel antipseudomonal agents to guide effective treatment and reduce further spread of high-risk clones.

## Data Availability

The datasets presented in this study can be found in online repositories. The names of the repository/repositories and accession number(s) can be found in the article/**Supplementary Material**. All draft genomes generated in this study were deposited in NCBI under BioProject PRJNA863458.
