# Supplementary material for: Whole genome sequencing of ceftolozane/tazobactam-resistant, XDR Pseudomonas aeruginosa ST773 in hospitalized critically ill infants and young children with ventilator-associated pneumonia
Source: Front Cell Infect Microbiol. 2026 Jul 10;16:1870600. doi: 10.3389/fcimb.2026.1870600 (PMC13417825; doi:10.3389/fcimb.2026.1870600)
Supplement: Supplementary file 1 [file DataSheet1.pdf]

Whole Genome Sequencing of ceftolozane/tazobactam-resistant, XDR *Pseudomonas aeruginosa* ST773 in hospitalized critically ill infants and young children with ventilator-associated pneumonia

Supplementary Table 1. Metadata of published *P. aeruginosa* genomes included in the phylogenetic analysis of the clinical isolates collected in the current study.

Strain	MLST	BV-BRC Genome ID	GenBank Accessions	Isolation Source	Collection Year	Isolation Country	Host Common Name
BA10747	773	287.29237	JARHLA000000000	blood	2022	India	Human
AZPAE14398	773	287.119	JTYQ00000000	itra-abdominal tract infection	2011	Germany	Human
AZPAE14889	773	287.1043	JTSZ00000000	itra-abdominal tract infection	2008	China	Human
AZPAE14959	773	287.974	JTQI00000000	itra-abdominal tract infection	2009	India	Human
PMM38	773	287.7402	NSSL00000000	nasopharynx	2004	South Korea	Human
MRSN110817	773	287.29448	JARSSE000000000	Perianal swab	2022	Ukraine	Human
AS_23	773	287.25792	JANAWV000000000	Petroleum contaminated soil	2019	Nigeria	NA
KE9524	773	287.16523	JABNNA000000000	rectal swab	2019	Germany	Human
BJ41	773	287.29758	JAPCVC000000000	sputum	2018	China	Human
60503	773	287.9848	CP041774	sputum specimen	2016	China	Human
PA-50010278	773	287.10398	WSYN00000000	surgical site	NA	USA	Human
KE9592	773	287.16522	JABNNB000000000	tracheal secretion	2019	Germany	Human
P20	773	287.29388	JAPWKV000000000	Urinary catheter	2021	Egypt	Human
P26	773	287.29384	JAPWKQ000000000	Urinary catheter	2021	Egypt	Human
P27	773	287.29386	JAPWKP000000000	Urinary catheter	2021	Egypt	Human
P30	773	287.29381	JAPWKM000000000	Urinary catheter	2021	Egypt	Human
P5	773	287.29404	JAPWLK000000000	Urinary catheter	2021	Egypt	Human
P8	773	287.29398	JAPWLH000000000	Urinary catheter	2021	Egypt	Human
15965	773	287.14274	JAFKQW000000000	urine	2017	Nigeria	Human
NCTC13715	773	287.8054	LR134330	urine	2011	United Kingdom	Human
P14	773	287.29394	JAPWLB000000000	urine	2021	Egypt	Human
PA790	773	287.14821	CP075176	urine	2019	India	Human
PSE6684	773	287.11536	CP053917	urine	2019	South Korea	Human
ST773	773	287.10162	CP041945	urine	2017	USA	Human
PS1	773	287.709	RHDU00000000	urine clinical sample	2018	Hungary	Human
ZYPA08	773	287.18479	JAIQLC000000000	Ward	2020	China	Human
85	773	287.4553	NXHS00000000	wound swab	2015	Ghana	Human
7	773	287.29813	JASEZX000000000	NA	2021	Nigeria	Human
21	773	287.29811	JASEZM000000000	NA	2021	Nigeria	Human

23	773	287.29808	JASEZK000000000	NA	2021	Nigeria	Human
35	773	287.29809	JASEZC000000000	NA	2021	Nigeria	Human
38	773	287.29807	JASEYZ000000000	NA	2021	Nigeria	Human
HPA0124	773	287.3301	CP137504	NA	2021	South Korea	Human
HPA0663	773	287.33007	CP137498,CP137499	NA	2021	South Korea	Human
HPA1346	773	287.33005	CP137493,CP137494	NA	2022	South Korea	Human
HPA2120	773	287.33003	CP137487,CP137488,CP137489	NA	2022	South Korea	Human
HPAS10	773	287.15745	JAHVND000000000	NA	2019	USA	Human

Supplementary Table 2: Genomic features of *P. aeruginosa* isolates investigated in the study

Isolate	Length (nt)	Sequencing depth	Contigs	Largest Contig	GC (%)	N50	ANI value	Completeness (%)	Contamination (%)	CDS	tRNA	tmRNA	rRNA
P1	6810415	9.45x	235	291454	66.07	74004	99.9558	99.68	0.21	6,239	62	1	5
P4	6894480	15.7x	270	175189	65.97	60950	99.9621	99.48	0.33	6,331	61	1	5
P13	6852361	6.32x	110	545839	66.03	197235	99.9511	99.68	0.21	6,331	61	1	5
P29	6902286	7.19x	199	296406	65.98	89756	99.958	98.97	0.21	6,330	61	1	5
P31	6915121	7.9x	189	267175	65.95	93687	99.9522	99.68	0.21	6,332	61	1	4
